# Passing from open to robotic surgery for dismembered pyeloplasty: a single centre experience

**DOI:** 10.1186/2193-1801-3-580

**Published:** 2014-10-03

**Authors:** Marcelo Di Gregorio, Andrei Botnaru, Laurent Bairy, Francis Lorge

**Affiliations:** Urology Department, Cliniques universitaires UCL Dinant-Godinne, Université Catholique de Louvain, 1 Av Gaston Thérasse, Yvoir, Belgium; Aenesthesia Department, Cliniques universitaires UCL Dinant-Godinne, Université Catholique de Louvain, 1 Av Gaston Thérasse, Yvoir, Belgium

**Keywords:** Pyeloplasty, Robotic surgery, UPJ obstruction, Laparoscopy, Minimally invasive surgery, Renal disease

## Abstract

**Background:**

The treatment of symptomatic uretropelvic junction obstruction (UPJO) has evolved towards minimal invasive endourologic and laparoscopic techniques. Robotic assisted laparoscopic pyeloplasty has achieved outcomes comparable to those corresponding to open and laparoscopic techniques.

The objective of this work is to demonstrate that the transition between open to robotic surgeries is straightforward. We analysed retrospectively “our initial results” in robotic assisted UPJ reconstruction procedures. Technical and convalescence aspects for 17 reconstructive robotic procedures performed by 2 surgeons in a 5 years period have been evaluated. Success consisted of no postoperative symptoms, no evidence of obstruction on mercaptoacetyltriglycine-3 diuretic renal scan or computed tomography (CT) and non-further treatment. Statistics: mean ± standard deviation, median and range.

**Findings:**

From 17 patients who underwent Da Vinci Robot procedure, 15 followed the complete treatment (2 were converted to laparotomy). Two patients had post-operative urine leakage; the stent was changed under sedation without further sequelae. The mean operative time was 189 minutes. The average hospital stay was 4 days. The average follow-up was 25 months. There was only one patient with UPJ stenosis at 6 months and he was treated by balloon dilation. All patients were followed with MAG 3 lasix renal scan, CT or urography. Except the patient with recurrent stenosis, all patients were asymptomatic without objective evidence of obstruction at the present time.

**Conclusions:**

Robotic pyeloplasty technique is feasible and gives good results without previous laparoscopic experience.

## Introduction

The ureteropelvic junction obstruction (UPJO) syndrome is a congenital or acquired disease. In the last 20 years the surgical approach has evolved radically. The open surgical technique has stayed the standard issue in UPJO for many years. Minimal invasive surgical options such endopyelotomy and classical or robotic laparoscopic pyeloplasty (CLP, RLP) were proposed as alternative treatments. During the nineties success with a rate of 50-88% (Motola et al. 1993; Gill and Liao 1998) for endourological procedures has been reported. However, endopyelotomy has a significantly reduced success and potential complications like critical bleeding (Albany et al. 2004).

Since Schuessler and co-workers reported in 1993 on the first laparoscopic pyeloplasty with similar result and lower morbidity to the “gold standard procedure” (open pyeloplasty) the dismembered laparoscopic classical pyeloplasty (DCLP) has become widely accepted for a treatment of this entity (Schuessler et al. 1993; Munver et al. 2004). The long-term results present a 90 to 95% of success (Klingler et al. 2003). Nevertheless it remains technically challenging because of its complexity and long learning curve. The main drawback of CLP is the relative difficulty of performing intracorporeal suturing that requires significant training and laparoscopic surgical expertise.

Nowadays many centres in the world have published their robotic surgical experience with the Da Vinci system (Intuitive Surgical, Inc, Sunnyvale, CA, USA) in the treatment of UPJO syndrome (Babbar and Hemal 2011). This approach makes easier the exact reproduction of an open surgical procedure in a new intracorporeal setting, giving the precise movement to performed laparoscopic reconstructive procedure. Da Vinci robotic system through computer enhancement gives to the surgeon an intuitive manipulation with magnified (X10) three-dimensional (3D) vision, increased degrees of freedom for surgical instruments that are maneuverable intracorporeally, tremor filtering and motion scaling. These characteristics give advantages like easiness of execution of intracorporeal suturing with better suturing and better overall operative time. The usual trend is to achieve robotic experience after having an extensive laparoscopic experience, as robotic systems become available in those institutions where surgeons are trained in laparoscopy. This trend is rapidly changing since more robotic platforms are more easily available.

We discuss the feasibility of the direct transition from performing an open to robotic assisted technique, without passing mandatory through laparoscopic surgery and having an expertise in laparoscopic surgery for dismembered pyeloplasty. In this frame, our institutional outcomes of robotic-assisted pyeloplasty are reported.

## Patients and methods

The data presented in this work involve a group of 17 patients who were treated by robotic assisted pyeloplasty in the urology department at Mont-Godinne Hospital from the Université Catholique de Louvain in Belgium. The seventeen patients diagnosed with UPJO syndrome underwent robotic assisted dismembered pyeloplasties between November 2008 and December 2012. This is an initial report in which we discuss our results and the advantage of robotic assisted surgery over other techniques available today.

**Figure 1:**
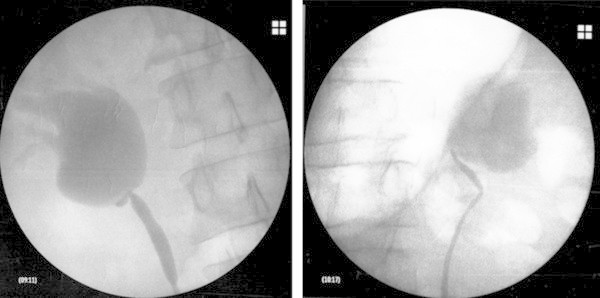
**Retrograde pyelography UPJ obstruction and hydronephrosis.** On the left side: the right kidney and on the right side: the left kidney.

*Clinical experience and data:* the patient's data were collected and presented in Table 1. All patients were evaluated before the surgical treatment by preoperative imaging techniques, either intravenous urography (IVU), or/and CT uroscan. All cases were confirmed by mercaptoacetyltriglycine (MAG-3) diuretic renal scintigraphy. Thirteen patients underwent a retrograde pyelography and ureteral double J stent placement before surgical procedure (Figure 1).

**Table 1:** **Demographics of patients**

Characteristic		Patients
Male/female		7/10
Mean age		51 ± 21
Median age (range) (years)		44 (17 – 77)
Body mass index (median)		24.18 ± 4.20 (24)
Affected side right/left		7/10
Patients with iterative procedure(s)		2
Patients with concomitant procedures		3
**Presentation based on clinical signs**		
Pain only	12	
Pain and hematuria	2	
Pain and pyelonephritis	3	
Pyelonephritis only	0	
Hematuria only Incidental imaging	0

**Figure 2:**
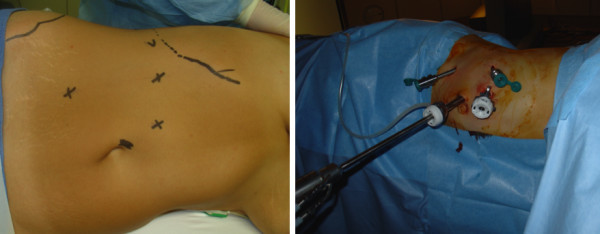
**Trocar placement.** On the left: landmark and on the right: trocar inside.

*Description of surgical procedure*: Firstly, a Foley catheter is placed and then the patient is placed in 45° lateral position, properly padded and secured to the operative table. Four transperitoneal ports are placed as such: a 12 mm port is placed near the umbilicus for the robotic Da Vinci stereoscopic camera, two 8 mm robotic ports are placed 30° cranially and caudally from the camera port (12 mm) spaced at 10 cm each other. A fourth 12 mm port is placed between the caudal and umbilical port. The last port is used by the assistant to help the dissection, suction, irrigation, introduction and removal of the sutures (Figure 2). The colon is reflected, the spermatic vessels, ureteric and ureteric-pelvic junction (UPJ) are exposed. The ureter is dissected caudally to the UPJ obstruction. The UPJ is excised caudally and the distal extremity is spatulated. If the renal pelvis is distended, a diamond-shaped wedge is excised and the pelvis is reduced and closed with running 4.0 or 5.0 Vicryl sutures. Placing a staying suture starts the UPJ anastomosis. We use 4.0 or 5.0 Vicryl for upper tract reconstruction and we adjust the suture to patient anatomy. We start the UPJ reconstruction by anastomosing the posterior wall. After this first suture, the proximal end of the double J ureteral stent is introduced in the renal pelvis. The anterior portion of the anastomosis is completed with the same suture. A double J ureteral stent is introduced percutaneously peroperatively for the patients who did not have preoperative stenting (three patients). A 7 – mm Jackson –Pratt or Redon drain is placed through a port side at the end of intervention. One patient had renal stone, which was treated with flexible ureteroscopy via one of the ports and stone basket extraction. Most of the patients were admitted overnight and discharged on the third postoperative day. The bladder catheter was removed two days after surgery and the ureteral stent was removed generally 6 week after the procedure.

## Results

**Figure 3:**
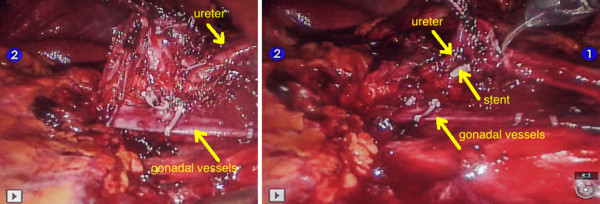
**Dissection difficulty.** On the left: pronounced scarring tissue with marked peri-ureteritis and on the right: ureter injury.

**Figure 4:**
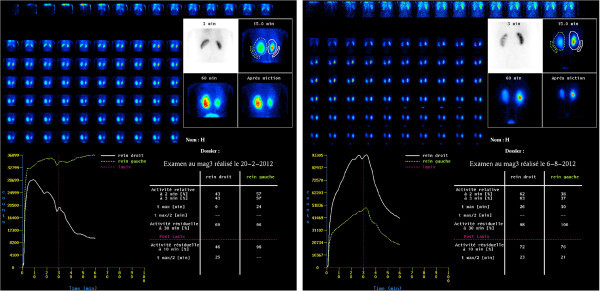
**99 mTc-labelled mercaptoacetyltriglycine (MAG-3) diuretic renal scintigraphy.** On the left: before surgery and on the right: after surgery.

**Figure 5:**
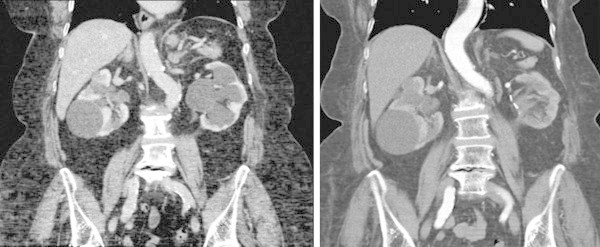
**CT scan.** On the left: left UPJ obstruction and hydronephrosis and on the right: disappearance of left hydronephrosis after surgery.

All patients had their UPJ treated laparoscopically with DaVinci robot assistance. There were two laparotomy conversions. The conversion was in both cases due to the difficulty to progress further with the dissection (Figure 3). They had previous kidney surgeries and marked fibrosis impending the dissection in the safest way. Surgeons experienced a difficulty in the progress of the procedure because of a pronounced scarring tissue in both cases and, indeed, an incapability of feeling the grasping force of dissecting instruments. Two other patients had post-operative flank pain and CT scan showed urine leakage. Both leakages were at the anastomotic site: one due to ureteral stent obstruction and in a second one by distal migration of the stent. The stent was changed under sedation without further sequelae in two cases with good results. The mean operative time was 189 minutes ± 40 (range from 120 to 257). In our department, assistants and fellows were involved in all cases, including robotic console time, which is mandatory for their training and this could account for some extra-time to the procedure. All cases, which required more than average 189 minutes operative time, were cases with previous renal procedures. The average blood loss was 14 ml ± 7 (range 10 to 30 cc) and the average hospital stay was 5 ± 3 days (range 2–16 days) with the median calculated at 4 days. A crossing vessel was present in 7 of 17 patients (41%). A reduction of redundant renal pelvis was performed in 14/17 patients (82%). One patient was treated concurrently for renal calculi, but was not cleared for stone burden and followed the ESWL. The analgesic requirement was minimal in the post-operative period. The average follow-up was 25 months (range from 3 to 49 months). The data are collected in Table 2. There was only one patient with UPJ stenosis at 6 months and treated by balloon dilation. The follow-up is too close in order to judge the effectiveness and the resolution of this annoying complication to the patient. Interestingly, 4 patients were treated after previous renal procedures: two had previous open pyeloplasties, one had partial nephrectomy and one had pyelolithotomy. Eleven patients were followed –up to 3 months with MAG 3 lasix renal **(**Figure 4) and CT (Figure 5) or IVU. Except the patient with recurrent stenosis all patients are asymptomatic without objective evidence of obstruction at this time.

**Table 2:** **Operative and postoperative findings**

Operative time min (median)	180
Decrossing of aberrant crossing vessels, (n°)	9
Drain removal, days (median)	1
Catheterization time, days (median)	1
Double pigtail removal, days (median)	54.6 ± 24.6 (46)
Hospital stay, days (median)	5.29 ± 3.04 (5)
Recurrence, n	1
Mean follow-up, month (median)	25.77 ± 16.54 (25)

## Discussion

Since the first reconstructive procedure for UPJO performed by Trendelemburg in 1886 (Singh and Hemal 2010), open pyeloplasty has been the standard treatment with success rates of 90-100% (Bird et al. 2011). Other possibilities of treatment as antegrade endopyelotomy or *Acucise* endopyelotomy have been described (OST et al. 2005). In 1993 conventional coelioscopic pyeloplasty (C-LPP) became a alternative for treatment with success rates comparable to open surgical repair with some advantages like reduced pain, less hospital stay, better cosmetic results and shorter convalescence (Hemal et al. 2010; Fallon et al. 2005). This technique was reserved to high volume centres with skilled laparoscopic surgeons owing good experience in laparoscopy (Inagaki et al. 2005). Comparison among open, endourologic and laparoscopic approaches to the obstructed ureteropelvic junction has been described in the literature (Brooks et al. 1995). Outcomes from robotic-assisted laparoscopic pyeloplasty have been reported by surgeons with experience in classical laparoscopic surgery (Venigalla et al. 2013; Sung et al. 1999). Robotic-assisted laparoscopy is presented as an innovative adjunct that makes surgeon’s tasks easy to perform and speed up the learning curve of laparoscopic technique (Bird et al. 2011; Uberoi et al. 2009). Robotic pyeloplasty has been adopted by most surgeons who have access to robotic systems, even in cases when the surgeon did not have previous laparoscopic experience (O’Brien and Shukla 2012). This is our case indeed.

The “fulcrum moment” is one of the great disadvantages of laparoscopic surgery, making the anastomotic step of the procedure one of the most difficult parts of the UPJ repair. When surgeons perform surgical manipulation through the laparoscope, their motor control system faces various challenges due to the “fulcrum effect” of the mechanical constraint at the incision point. These challenges include inversion and scaling of movements, altered sensation of forces due to mechanical advantage and friction at the incision point (Westebring-van der Putten et al. 2008). The robotic technology takes away this contra intuitive and hard-to-learn laparoscopic skills by improving the laparoscopic haptic perception. Furthermore, it provides the novice surgeon with a new scaling system that allows progressing rapidly. On the other hand, laparoscopic surgery is a technique requiring extensive training (Gallagher and Satava 2002).

The first limitation of laparoscopic UPJ repair is uncomfortable ergonomics. Surgeons are required to stand in uncomfortable positions and holding long instruments. Medical robotics allows the primary operating surgeon to sit while operating, and provides armrest. This significantly improves the primary operating surgeon’s ergonomics, and thus comfort. Medical robotics facilitates the surgeon’s ergonomics even further, by means of a computer interface between the surgeon’s hands and the instrument tips. This translates the natural/intuitive movement of the surgeon’s hands into the desired movement of the robotic instrument, bypassing the handle and shaft of the laparoscopic instrument (Stylopoulos and Rattner 2003).

The robotic systems, however capable of enhancing the surgeon performance of a wide variety of tasks, they cannot replace the surgeon’s problem-solving ability. Instead, they will redefine his role by providing their complementary capabilities and an ergonomically efficient and more user-friendly working environment (Stylopoulos and Rattner 2003).

Robotic technology provides the means to overcome many of the limitations of minimal invasive surgery. This is accomplished in four ways. First, the robotic instruments themselves have five to seven degrees of freedom of movement compared to the four degrees of freedom of movement in traditional laparoscopic instruments (Ballantyne and Moll 2003). Second, the computer in the robot eliminates the fulcrum effect. Third, the robotic computer is also programmed to filter out the physiologic tremor in the human hand, which can be greatly magnified at the end of a very long instrument. Finally, robotic computers allow the surgeon to choose to scale, either up or down, the ratio of the size of the movement of his or her hands to the movement at the instrument tips (Ballantyne and Moll 2003).

There were studies comparing robotic pyeloplasties (RP) and laparoscopic pyeloplasties (LP). It was found that the procedures had similar outcomes and surgical training had a significant impact on the outcomes (Weise and Winfield 2006). The robot helped the surgeon to do a precise dissection, excising the flap, suturing the anastomosis faster and in more relaxed condition. A recent meta-analysis of articles published in the literature about RP versus LP reveals that over the past 8 years, RP has been successfully performed worldwide, and it is a minimally invasive procedure that is safe and effective, with results that are as good as, or better than, the results of open surgery or LP (Braga et al. 2009). The robotic surgery has advantages like 3-D high-definition optics, magnification, wristed instrumentation and tremor control that provide a quality dissection and anastomosis, especially if a stent is placed previously (Ferhi et al. 2009). Retroperitoneal and transperitoneal approaches are possible (Cestari et al. 2010). There are no currently accepted definitions of what constitutes a difficult case for RP. It appears that RP can be applied to almost all patients with UPJ Obstruction (Lucas and Sundaram 2011; Hemal et al. 2008). A recent multi –institutional study identified crossing vessels and previous endopyelotomy as a factor that might be associated with decrease success rates (Singh and Hemal 2010; Sivaraman et al. 2012). We have two conversions to open pyeloplasty, but they were related to difficult dissection caused by very fibrotic and inflammatory tissue.

Nevertheless robotic pyeloplasty is a feasible alternative to laparoscopic pyeloplasty, at the present moment the cost is a clear difficulty to adopt it. The surgery cost is about three times the cost of an open classical procedure. This is due to the expensive disposable material and its maintenance. In addition, not all the health care systems are willing to reimburse all robotic procedures (Shah et al. 2007). One may argue about the real convenience involving robotic surgery and all new minimal invasive approaches versus the gold standard treatment. However, the shorter admission stay is making this technique very attractive for the patients and insurance companies. There is no cost-efficiency study yet done for the robotic pyeloplasty in Belgium or in our institution.

As it comes obvious from a historical point of view, all innovations and new technologies are more expensive at the beginning of their production. Habitually other factors than high cost (about $1.8 million USD) as difficulty of operation and the inability to routinely manage an operation prevents from the quick adoption of innovations. In our institution the first robotic Da Vinci system was acquired in 2007 and this was possible through our non-profit foundation. Robotic activity is also sponsored though national financing program, which leaves the freedom to each university hospital in Belgium to decide the priorities for development of expensive technologies.

## Conclusions

Robotic assisted laparoscopic pyeloplasty for the correction of ureteropelvic junction is feasible without previous training experience in laparoscopy and it has similar outcomes. Robotic assistance allows the transition from open to laparoscopic procedure without difficulty, making easier the dissection and intracorporeal suturing. This is due to the intuitive characteristics of robotic technology. Robotic pyeloplasty needs a low learning curve for a minimal invasive reconstructive surgery. Laparoscopic experience is a plus but not “*a must”*, even if it helps the surgeon in performing difficult procedures. We feel that *laparoscopic technique will be replaced by robotic technique* in UPJ reconstructive procedures as laparoscopy faded in favour of robotic technique in most institutions where robotic surgery is available for radical prostatectomy.

### Consent

“Written informed consent was obtained from the patient’s guardian/parent/next of kin for the publication of this report and any accompanying images”.
